# From signal to bedside: the missing link between ECG-AI explainability and clinical trust

**DOI:** 10.1093/ehjdh/ztag092

**Published:** 2026-06-11

**Authors:** Akinchan Bhardwaj, Ishita Vasudev, Pushkala Jayaraman

**Affiliations:** Department of Cardiology, Kauvery Heart Institute, 200 Feet Radial Road, Kovilambakkam, Chennai 600129, India; Department of Internal Medicine, BronxCare Health System, Icahn School of Medicine at Mount Sinai, NewYork, NY, USA; The Windreich Department of Artificial Intelligence and Human Health, Icahn School of Medicine Mount Sinai, NewYork, NY, USA

Arends *et al*.^1^ recently evaluated 12 attribution methods applied to deep neural network-based ECG classifiers across more than 1.5 million attribution maps. Their systematic assessment of inter-method similarity, self-consistency, dependence on model weights, and perturbation sensitivity leads to a significant conclusion: current attribution techniques exhibit limited reliability and should be used with caution in high-stakes clinical settings. While these conclusions are well-supported, an additional critical dimension warrants further discussion because it was not fully addressed in the study.

The gap identified by Arends *et al*.^1^ reflects not only a technical limitation but also a fundamental challenge in translating explainability into clinically meaningful decision support. Comprehensive evaluation of explainable AI in healthcare should encompass three distinct evidentiary layers: predictor performance, explanation faithfulness, and clinical decision impact. Although Arends *et al*.^1^ rigorously address explanation faithfulness, the effect on clinical decision-making remains largely unexplored.

Several findings warrant particular attention. Attribution methods demonstrated only moderate self-consistency across model initializations (mean Pearson correlation of 0.41–0.65), showed only partial dependence on learned model parameters, and varied substantially across methods.^1^ Even widely used approaches such as GradCAM showed limited performance degradation during perturbation testing, indicating weak correspondence between highlighted regions and the features that actually drive model predictions.^1^ These limitations are not minor technical imperfections. Such shortcomings may result in clinicians encountering visually convincing heatmaps, such as those highlighting the *P*-wave in atrial fibrillation or the ST segment in ischaemia, without assurance that these regions accurately reflect the model's reasoning.

This disconnect has direct clinical implications. Usability studies indicate that attribution maps are perceived as only modestly helpful, with average usefulness scores of ∼0.66 on a −3 to +3 scale.^2^ In clinical medicine, a tool that is marginally helpful yet potentially misleading is not neutral; it actively risks misguiding clinical judgment. When a cardiologist reviews an AI-generated attribution map, it remains unclear whether this appropriately calibrates diagnostic confidence or introduces anchoring bias. Similarly, the impact on downstream decisions, such as whether to admit, investigate further, or escalate therapy, remains uncertain. These empirically testable questions have yet to be adequately addressed.

This gap underscores a broader conceptual tension within the field. Current frameworks define explanation quality in terms of interpretability, parsimony, and fidelity, which are primarily evaluated from an engineering perspective.^3^ In practice, however, explanation is inherently contextual. An attribution that is internally consistent and mathematically rigorous may still fail to provide meaningful guidance in a time-constrained clinical environment. Post hoc methods can create an illusion of understanding at the level of individual decisions.^4^ Visual explanations may appear intuitively convincing even when they do not accurately reflect model reasoning, increasing the risk that clinicians over-trust AI outputs based on explanations that are visually plausible but mechanistically unreliable.^4,5^

Certain methodological constraints of the study also need to be considered. The analysis relied on single-centre data and a single model architecture (ResNet-based CNNs), with heuristic hyperparameter tuning and no external validation. Whether the observed attribution instability is intrinsic to these methods or partly reflects dataset- or architecture-specific characteristics remains an open question. Transformer-based or graph neural network architectures, as well as the generative counterfactual approaches recommended by the authors, warrant parallel evaluation.

The next phase of ECG-AI research should move beyond technical validation of attribution methods to include structured evaluation of their clinical impact. Randomized reader studies comparing clinician performance with AI predictions alone vs. AI supplemented with explanations, assessing diagnostic accuracy, confidence calibration, and error override rates, would directly test whether explanations are beneficial or detrimental. Workflow-based A/B evaluations could similarly determine whether attribution maps meaningfully influence management decisions. Reporting standards for clinical AI studies would benefit from an explicit distinction between explanations validated against technical criteria and those evaluated for clinical decision-making impact, to ensure that visual plausibility is not mistaken for clinical utility. Arends *et al*.^1^ ask whether current attribution methods produce signal or noise. Their data indicate that, at the level of model explanation, the answer is often closer to noise. The more consequential issue is whether clinicians can reliably distinguish between the two at the bedside. Until this challenge is addressed, attribution-based explainability risks creating the appearance of understanding without substantive insight.
